# Spectral Interferometry with Electron Microscopes

**DOI:** 10.1038/srep33874

**Published:** 2016-09-21

**Authors:** Nahid Talebi

**Affiliations:** 1 Stuttgart Center for Electron Microscopy, Max Planck Institute for Solid State Research, Heisenbergstr. 1, 70569 Stuttgart, Germany

## Abstract

Interference patterns are not only a defining characteristic of waves, but also have several applications; characterization of coherent processes and holography. Spatial holography with electron waves, has paved the way towards space-resolved characterization of magnetic domains and electrostatic potentials with angstrom spatial resolution. Another impetus in electron microscopy has been introduced by ultrafast electron microscopy which uses pulses of sub-picosecond durations for probing a laser induced excitation of the sample. However, attosecond temporal resolution has not yet been reported, merely due to the statistical distribution of arrival times of electrons at the sample, with respect to the laser time reference. This is however, the very time resolution which will be needed for performing time-frequency analysis. These difficulties are addressed here by proposing a new methodology to improve the synchronization between electron and optical excitations through introducing an efficient electron-driven photon source. We use focused transition radiation of the electron as a pump for the sample. Due to the nature of transition radiation, the process is coherent. This technique allows us to perform spectral interferometry with electron microscopes, with applications in retrieving the phase of electron-induced polarizations and reconstructing dynamics of the induced vector potential.

Time−frequency analysis is a well-known methodology in signal processing. It provides us with unprecedented knowledge about the response of systems in time−energy phase space^1^. In fact, many characteristics of extremely short emitted laser pulses can only be retrieved by means of ultrafast characterization techniques such as interferometry, and cannot be obtained by simple Fourier-based spectroscopy techniques. Besides that, curiosity about the dynamics and the rich physics happening on the nanoscale^2^ gave rise to the field of ultrafast nanoscience, using either optical excitations^3^,^4^, X-rays^5^, and most recently electron probes^6^,^7^,^8^,^9^,^10^,^11^.

Among all these, the last technique can provide us with the best possible spatial resolution, because of the short de-Broglie wavelength of the electron wave packets. Electron microscopes with pulsed electron sources are at the heart of ultrafast techniques. Photoemission electron sources, combined with state-of-the-art aberration-corrected magnetic lenses, have paved the way towards time–space characterizations of sample responses with sub-picosecond time resolution and nanometer spatial resolution^12^,^13^. Another advantage of electron probes comes from the ultrafast interaction of the relativistic electrons with the sample, so fast that a broad excitation bandwidth is imposed, from sub–electronvolt (eV) energies to kilo eV^14^. Especially when the spectroscopy techniques are considered, two operational modes of electron microscopes, namely cathodoluminescence (CL) and electron energy-loss spectroscopy (EELS), provide fast characterization of materials with perfect spectral and spatial resolutions.

Regardless of all these advantages, electron-based time–energy characterization techniques are still far from competing with optical methodologies like spectrography and spectral spectrometry^3^. Despite the fact that spatial holography was first introduced within the context of electron microscopy^15^, and despite all the successes that holography has brought about^16^,^17^,^18^, applications of the interference patterns in electron-based techniques have been so far limited to the spatial domain. This is mainly because of the fact that full control of the intensity, the time-duration and the coherence of optical pulses became possible many years ago with the introduction of lasers, whereas still intensive work is carried out to provide equivalent electron sources^9^,^19^,^20^. Moreover, all the research in this field is still based on the photoemission process and the synchronization of the emitted electron probes with a pumping laser beam. Because the photoemission process sustains an inherent time jitter of a few femtoseconds, the technique suffers from a fundamental temporal broadening which is larger than the coherence time of the single electron wave packet^21^,^22^. In order to avoid space charge forces, single-electron pulses are routinely exploited. However, an ensemble of electron pulses, composed of at least 10^7^ incoming electrons, is needed to probe the dynamics with an acceptable signal-to-noise ratio. The whole effective electron pulse duration is known to be affected by the statistics of the electron pulses in this ensemble, with respect to the time reference which is dictated by the laser (see Fig. 2 of ref. 22). Considering the quantum superposition of the electron pulses, an environmentally included selected eigen-basis in Dirac notation would be necessary to precisely describe this electron pulses ensemble in the electron-laser pump-probe spectroscopy techniques, which is beyond the scope of the present manuscript. In other words, time-jitter directly influences the effective time-broadening of the electron pulses. This effect prohibits the electron-laser pump–probe techniques to be used for spectral interferometry, for which a temporal broadening at the level of attoseconds is ideally required. Moreover, understanding dynamics of photoemission processes, investigation of Bloch wave packets, electron motions in chemical reactions, and dynamics of catalysts demands such time resolution^23^.

## Results and Discussions

### Electron-driven photon source

However, it is not necessary to trigger electrons with photons. Another approach, which is proposed here is to trigger the photon trajectories with electrons. Relativistic electron beams support various mechanisms of radiation, e.g. Cherenkov radiation^24^, Smith−Purcell radiation^25^,^26^, and transition radiation^27^, to only name a few. The Smith−Purcell effect is commonly used to design free electron lasers in the THz frequency range^28^,^29^. Because of the interaction of the electrons with gratings rather than with a single scatterer, coherent electromagnetic radiation is formed in the far-field by the interference of the transition radiation from each individual element in the grating in a phase-matched way. Considering that each scatterer reradiates a single photon in interaction with the electron, certainly more than two photons, and hence a certain number of grating elements, is required to form an interference pattern in the far-field^30^.

Considering the above-mentioned mechanisms of radiation, an electron–based spectral interferometry technique is proposed here, which can be simply integrated with the current state-of-the-art electron microscopes. The idea is to use coherent transition radiation in an interaction of the electron with a structure, to force each single electron to produce its own conjugate photon pulse, and hence its own time reference. It should be mentioned that although time-resolved cathodoluminescence experiments with picosecond time resolutions have been already demonstrated^31^, there is still no report on attosecond time resolution or reconstruction of the spectral phase of samples using electron microscopes.

**Figure 1:**
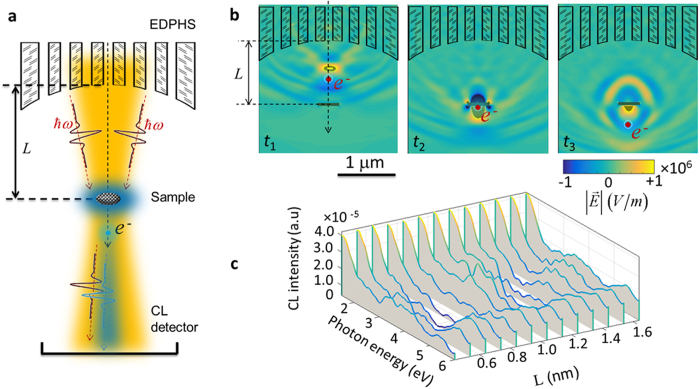
The concept of Spectral interferometry with electron microscopes. (**a**) The setup is composed of an EDPHS, which in interaction with a swift electron generates photons which are scattered in a unidirectional way towards the sample and focused on it. (**b**) The EDPHS excitation interferes with the electron induced CL emission of the sample, either destructively or constructively, depending on the distance L between the sample and EDPHS. The sample is positioned at the focal point of the EDPHS. (**c**) Calculated CL spectra versus distance and energy, where the shift of resonances in the energy–distance map due to the interference is apparent. The sample here is a silicon disc with a thickness of 40 nm and a radius of 150 nm.

An electron-driven photon-source (EDPHS) is considered here. To propose a technique which can be easilly incorporated into an electron microscope, a mesoscopic metamaterial-based EDPHS has been designed. Recently, it has been demonstrated that metamaterials, in interaction with relativistic electrons, can radiate coherent optical beams^32^. However, the metamaterial-based EDPHS proposed here, is designed to generate coherent and focused transition radiation along the electron trajectory, as shown in Fig. 1a. The emission should be unidirectional, so the generated photons are focused on the sample, instead of being scattered in a huge angular range at far-field, as is schematically shown in Fig. 1b. The criteria for the design of this EDPHS and the functionality and characteristic behaviors of the proposed EDPHS will be presented later.

The emission from the EDPHS will propagate at the speed of light, which is faster than the electron speed. However, due to retardation, it will take a few femtoseconds for the generated photons to leave the EDPHS. We use this fact to control the arrival time of the electron and the EDPHS radiation at the sample position by changing the distance between the sample and the EDPHS (Fig. 1b). A characteristic delay is thus assigned to the photon pulse with respect to the electron as 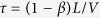 where *V* is the electron speed and *β* = *V*/*c* is the speed of the electron normalized to the speed of light (*c*). *L* is the distance between the EDPHS and the sample.

It is well known that transition radiation is mutually coherent with the evanescent field of the relativistic electron that has created it^33^. Moreover, since the dissipative loss is well controlled in the EDPHS, the generated photons are converted into the radiation continuum and focused upon the sample. In such a design, the electron-induced polarization in the sample interferes with the EDPHS radiation, where this interference phenomenon can be either constructive or destructive, depending on the distance *L* between the EDPHS and the sample. This interference pattern becomes evident in both EELS and CL spectra. The focus in this paper will be on the CL detection method, with which the role of interference is more apparently captured at the far-field, since the nonradiative and dissipation losses do not contribute to the CL spectra^34^,^35^.

Theoretically speaking, the CL spectrum is interpreted as the number of photons emitted per unit of solid angle emission 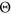 and per unit of photon frequency *ω* to the far field, as 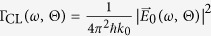, where 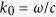, 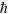 is the Planck constant, and 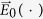 is the amplitude of the scattered electric field in the far-field region^33^. Since the scattered field has contributions from both electron-induced polarization in the sample and also the EDPHS radiation, the CL spectra can be explicitly written as:


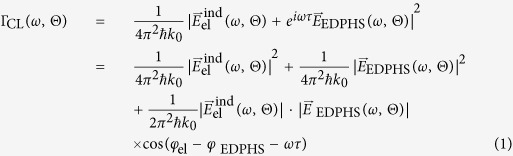


where 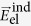 and 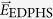 are the electric-field contributions induced by the electron and EDPHS excitations, respectively. Equation (1) clearly indicates that, similar to in any kind of interference phenomenon, the spectral phases 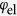 and 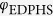 due to the electron and EDPHS excitations are encoded in the overall intensity. We observe a maximum intensity whenever 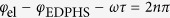, where *n* is an integer. Moreover, controlling the delay (*τ*) by simply changing the distance, we can record a full interference pattern in the time–energy (*τ* − *E*) phase space (Fig. 1c).

**Figure 2:**
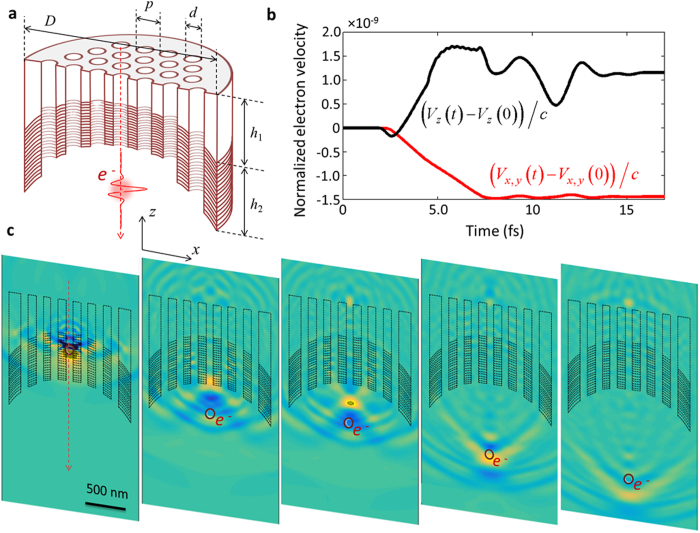
Time domain response of the EDPHS. (**a**) Topology of the EDPHS, which is composed of an inverted superlens with an incorporated void hexagonal photonic crystal, *h*_1_ = 400 nm, *h*_2_ = 600 nm, *D* = 1.6 μm, *d* = 100 nm and *p* = 200 nm. (**b**) Computed modulation of the relative electron velocity (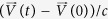, where *t* is time, 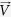 is the electron velocity, and c is the speed of light) due to the interaction with EDPHS. (**c**) *z*-component of the electron–induced electric field at times depicted in each frame.

The fundamental requirements for an efficient EDPHS that can be used for the application considered here are 1^st^- the EDPHS should couple the electron-induced polarization to the far-field radiation, 2^nd^- the EDPHS should exhibit a unidirectional focused radiation on the sample, and 3^rd^- the EDPHS should manifest a broad-band far-field spectrum, to investigate the ultrafast responses of the sample with high efficiency. To meet these criteria, the concomitant incorporation of photonic crystal and of metamaterial concepts is considered here. First of all, for a mesoscopic EDPHS with the ability to focus the transition radiation, an inverted superlens is introduced. This multi-layered structure is composed of Ag/Al_2_O_3_ thin films, each with a thickness of 30 nm, positioned upon a spherical plano–concave silica lens (see Fig. 2a), with overall thicknesses of *h*_1_ = 400 nm, *h*_2_ = 600 nm and a radius of curvature of the lens of 1000 nm. In an effective medium approximation, the multi-layered metal/dielectric structure demonstrates an electromagnetic hyperbolic behaviour. This behaviour exhibits a huge photonic density of states and hence an enhanced coupling efficiency of the quantum emitters to the optical modes. Moreover, a void hexagonal photonic crystal is also incorporated into the lens. The collective excitation of a photonic crystal lattice can generate a better coupling of the excited beams to the radiation continuum, as will be demonstrated later. This behaviour is especially appealing here to prohibit the activation of non-radiative de-excitation processes.

To understand the interaction of a swift electron at a kinetic energy of 200 keV with the EDPHS, a fully self-consistent numerical approach within the frame work of combined Maxwell and Lorentz equations has been performed, with the details being pointed out in the Methods section. The interaction of the relativistic electrons with nanostructures and thin films can be well explained by the undepleted pump approximation [32]. However, the amount of recoil that the electron receives is illustrative (Fig. 2b and Fig. S1) and demonstrates well the intensive radiation from the EDPHS. The time reference is set here as the time that the electron enters the simulation domain. With the time interval of (2.4 fs, 4.4 fs), the electron traverses the silica substrate, then at *t* = 7.0 fs it leaves the EDPHS nearfield region. Interestingly, the modulation of the electron velocity is even more apparent during the time interval 7 fs to 14 fs, when the electron is still in the wake of the EDPHS emission. The strongest recoil that the electron receives is at *t* = 11.3 fs, when the electron reaches the focal point of the plano–concave lens.

The time domain simulations perfectly demonstrate the temporal response of the EDPHS (Fig. 2c). The emission from the EDPHS is in the form of an ultrashort positively chirped few cycle pulse (see Supplementary Fig. S2). The radiation is quite intense near the focal point, which is approximately 900 nm away from the EDPHS and along the electron trajectory and, interestingly, catches up to the electron exactly at this point. The radiation spreads very fast overtaking the electron after the focal point.

**Figure 3:**
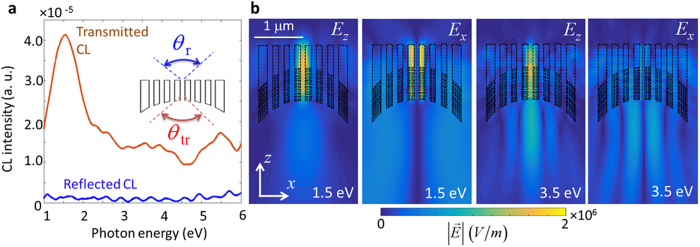
Frequency-domain response of the EDPHS. (**a**) Calculated CL spectra at the detector position above and below the EDPHS. The solid angles of the detectors are taken as 1.84 sr. 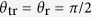. (**b**) Magnitude of the Fourier-transformed electric field components at the energies depicted in each frame.

To understand the spectral features of the emission from the EDPHS, the calculated CL spectrum is shown in Fig. 3a, for which the emission is divided into the transmitted and reflected contributions. This splitting, as well as the computed Fourier-transform of the electric field components (see Fig. 3b) demonstrates the unidirectional behaviour of the emission, and also the focusing capability of the superlens. The polarization state of the EDPHS emission is also understood from these figures. The field profiles very much resemble the first-order (LG11) and second-order (LG21) transverse magnetic Laguerre-Gaussian optical beams, at *E* = 1.5 eV and *E* = 4.0 eV, respectively. Moreover, the peak intensity of the EDPHS emission at the focal plane is 2.65 W/cm^2^. Interestingly, the intensity is exactly at the level at which an interference effect in EELS should take place^11^.

### Spectral Interferometry Technique

**Figure 4:**
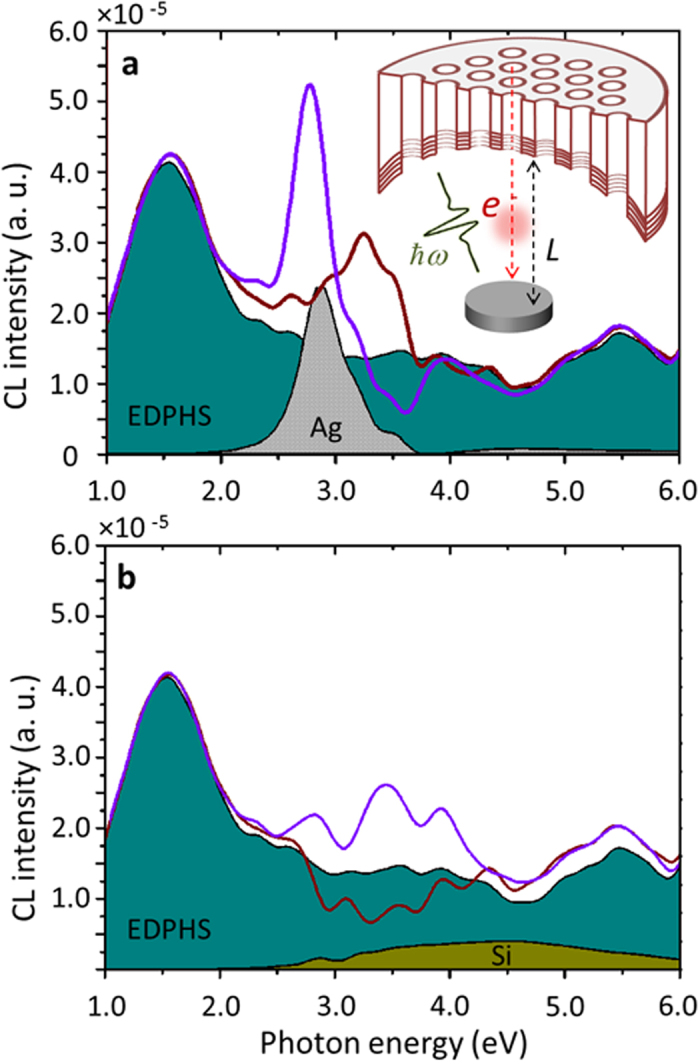
Calculated CL spectra. CL spectra for an isolated EDPHS (teal shadowed region) and also a sample coupled to EDPHS at the distances *L* = 1.0 μm (violet line) and *L* = 1.3 μm (red line) between the sample and EDPHS, for (**a**) a silver disc and (**b**) a silicon disc as the sample. The CL spectra for individual Ag and silicon discs are shown with grey and olive shadowed regions, respectively.

A sample positioned along the electron trajectory will interact with both the electron and the EDPHS excitations. To investigate the functionality of the proposed method for analysing the ultrafast responses of different samples, an Ag disc and a Si disc both with 300 nm diameter and 40 nm thickness, are used here as samples. The CL spectra of the individual Ag sample and EDPHS structure are shown in Fig. 4a, by grey and green shadowed regions, respectively. Because of the excitations of plasmons in the silver disc, the CL emission from the sample is intense and a clear peak is observed at *E* = 2.9 eV. When both the EDPHS and the sample are positioned along the electron trajectory, they form coupled structures with an overall CL spectrum that is not just the sum over the CL spectra of the different elements. Since the emissions from the EDPHS and the sample are mutually coherent, the interference between the EDPHS and the electron-induced emissions from the sample can alter the CL spectra significantly, depending on the distance *L* between the sample and EDPHS. For the case of the Ag disc chosen as the sample, the changes in the CL spectra are limited to the energy band of 2.0 eV to 3.0 eV. This is due to the fact that the plasmon-induced cathodoluminescence is rather narrow band. This is quite different from the case when the sample is a Si disc, for which the CL emission of the sample is very broad band (Fig. 4b). Hence fluctuations in the overall CL spectra of the coupled EDPHS and sample structure also cover a huge energy range, from 2.0 eV to 6.0 eV.

A full interference pattern in the CL energy–distance map becomes apparent, when a series of CL spectra is acquired at preassigned distance steps. A fine sweeping of the distance can be well accomplished by state-of-the-art piezoelectric actuators. Considering this, the proposed methodology can be used to access the distance/delay axis with enough sampling points, to precisely characterize the temporal evolution of the responses according to the Shannon sampling theorem.

**Figure 5:**
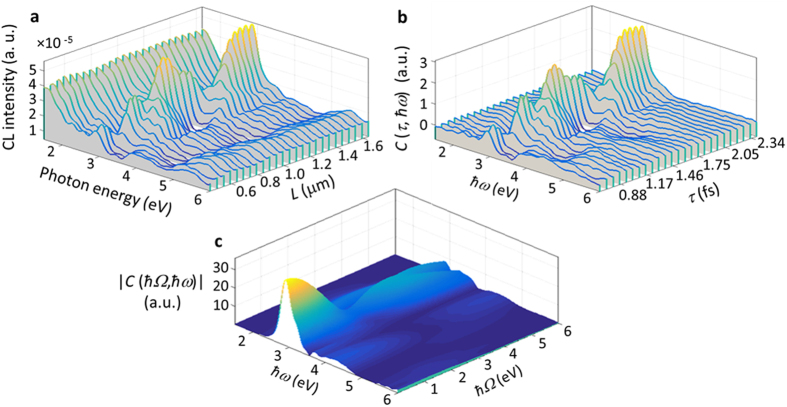
Spectral interferometry using the energy-distance CL map. (**a**) Series of calculated CL spectra of the coupled system of EDPHS and a silver disc positioned at a distance *L* from EDPHS, where the distance is swept by steps of 50 nm. (**b**) The extracted energy-time correlation function (see Eq. (2)). (**c**) The Fourier-transformed two-frequency correlation function.

It will be shown in the following how the proposed methodology can be used to retrieve the spectral phase. The calculated CL spectra for the coupled system of EDPHS and silver disc are shown in Fig. 5a, versus distance and energy. The distance axis is sampled at intervals of 50 nm, and the whole range is from 0.42 μm to 1.62 μm. The spectral intensity which is measured at the first step, without using the phase retrieval algorithm, is the CL from the whole setup, which includes CL from individual EDPHS and samples, as well as the interference between the emissions from these two structures. This fact is comprehended from eq. (1). The purpose of the exploited spectral interferometry is to recover intensity and phase of the CL spectra from the sample. Moreover, Fig. 5a, clearly demonstrates that, the whole CL spectrum is not only a slight difference to the EDPHS spectra, neither an accumulative spectrum of individual EDPHS and sample spectra. Both EDPHS and sample transition radiations, as well as the interference between those radiations at the CL detector, are responsible for the overall CL spectra.

To retrieve the spectral phase, a reference signal is required, which is taken here as the emission the EDPHS structure. According to the Fourier-transform spectral interferometry^3^, even one single CL spectra of the coupled EDPHS/sample system at a precise distance/delay would be enough to retrieve the spectral phase. However, using the full energy–distance map allow better noise rejection^36^. A method is proposed here that is suited to retrieve the spectral phase of an ultra-broadband electron-induced signal, within 2 eV to 6 eV energy range.

The first step is to extract a correlation function from the CL map. Taking the EDPHS emission as the reference, the energy–distance and its corresponding energy–time correlation map is given by:


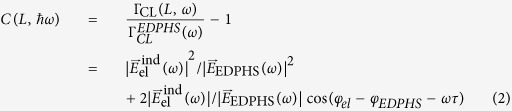


where 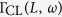 is the energy–distance CL map shown in Fig. 5a and 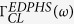 is the CL spectrum of the EDPHS. This correlation function is different from the generally used degree of first-order coherence^37^, and is only introduced here to acquire the spectral phase with high accuracy. To do this, we first map 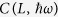 to the time–energy correlation 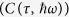 by simply using the delay–distance relation 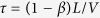 (see Fig. 5b). 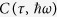 can be then transformed into the energy-energy correlation function 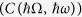, as


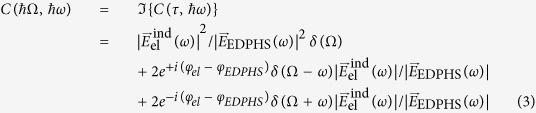


where 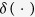 is the Dirac-delta function. It is already apparent here that fine-sweeping of the delay axis as well as covering at least few oscillation cycles is required to be able to take the Fourier-transform. 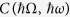 (see Fig. 5c) is readily used to retrieve the spectral intensity and phase by integrating it over the positive frequencies. The retrieved electric field spectra can be expressed as:


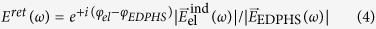


**Figure 6:**
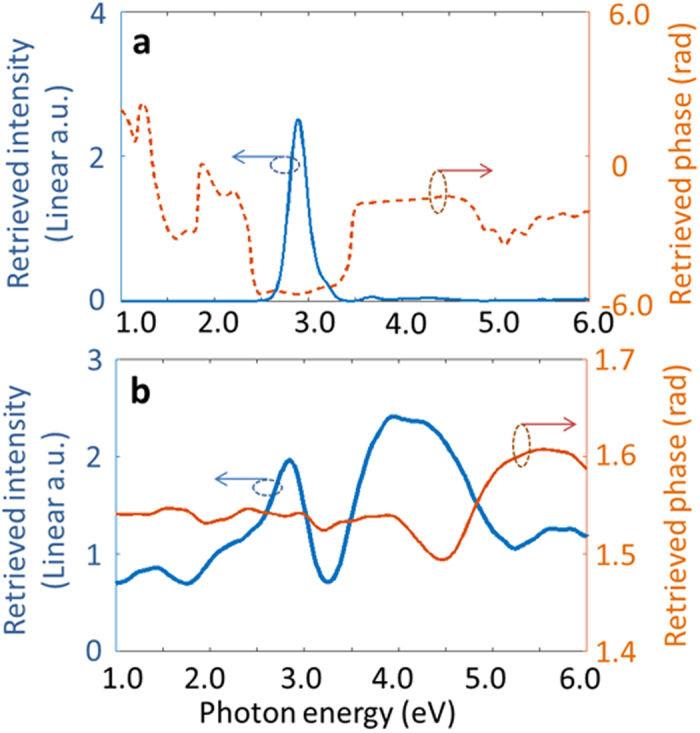
Reconstructed spectral intensity and phase. The retrieved electron-induced electric field (**a**) in a silver disc and (**b**) in a silicon disc, for an electron at the kinetic energy of 200 keV penetrating through the center of the disc. Each disc has a diameter of 300 nm and thickness of 40 nm.

It is apparent from Eq. (4) that the proposed technique can provide us with a direct electric field reconstruction. However, it is the ratio of the electron-induced field to the EDPHS field that is actually retrieved. In this regard, the retrieved phase is the phase of the electron induced emission (transition radiation) from the sample, relative to the phase of the EDPHS radiation. This approach is clearly a differential measurement. In other words, the experimental characterization of the EDPHS electric field with an optical interferometry technique like SPIDER would be highly beneficial. Doing so, the retrieval algorithm presented here will provide us with direct access to 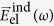. Hereafter, only the results of the phase retrieval algorithm which leads to eq. (4) are presented. The retrieved spectral phase and intensity are shown in Fig. 6a,b for both Ag and Si discs, respectively. Within the frequency range of 2.0 eV to 3.0 eV the differential intensity and phase of the electron-induced electric field in the silver disc can be retrieved with a high accuracy, thanks to the rather flat spectral intensity of the EDPHS in this frequency range, as is understood by comparing Fig. 6a to Fig. 4a. Even the ultrabroadband electron-induced resonances of the silicon disc are captured with good accuracy.

Although the presented EDPHS would serve as a highly efficient photon source for the presented interferometry technique, other electron-driven photon sources which directly employ quantum emitters and active regions can be also used for this purpose. Collective responses of atoms and quantum dots to the electron beam might also produce a superradiance continuum^38^,^39^, and a combination of these with metamaterials would lead to enhancement of the quantum efficiency and unidirectional emission.

Furthermore, there is no fundamental reason why the interference pattern should not be captured by the EELS detector, besides the fact that the EELS spectrum has direct contributions from loss channels such as dissipation and nonradiative de-excitations. A suitable design for the EDPHS can also overcome these problems, by incorporating superradiance combined with metamaterials to control the directionality of the emission.

In an adiabatic Wolkow approximation^40^, the electromagnetic vector potential is accumulated in the phase of the electron wave-function. An EELS or CL spectra provide us with knowledge about the probability of the electron to emit certain photons, or the intensity of the emitted photons to the far-field. In this regard the phase of the electron wave-function, and therefore the accumulated vector potential, is neglected. Spatial holography technique can retrieve the spatial phase of the electron wave-function^16^. The proposed spectral interferometry technique here can be used to characterize the vector potential in time, energy, and space. As an example, it provides a direct access to the spatiotemporal multipolar resonances of nanostructures, which are not accessible in routinely exploited EELS or CL measurements. Although spectral features of such resonances are captured in CL or EELS measurements, always electrodynamics simulation methods are needed to clarify the physics behind each resonance, by retrieving the real-space spatiotemporal distributions of the fields^41^,^42^. Exploiting a pulse characterization technique such as the spectral interferometry technique would be necessary to experimentally retrieve the spatiotemporal phase and oscillations of the electron-induced excitations in the sample.

The EDPHS structure proposed here emits a coherent radiation within the energy range of 1 eV to 6 eV. In this regard, time-frequency responses of metamaterial elements, plasmons, photonic crystals, and quantum optical fluctuations of quantum dots, nanoemitters, atoms and molecules can be probed. In principle, depending on the EDPHS radiation, this technique may also be applied to higher photon energies, by incorporating coherent Bremsstrahlung for example^43^. Moreover, if the proposed state-of-the-art Piezo–stages are combined with hybrid manipulators, even a better control of the distance between the EDPHS and sample can be achieved, and hence a wide dynamic range for characterizing the temporal behaviours will be addressable. In fact, a hybrid 3-axis manipulation system and a precisely designed sample holder should be considered as an additional counterpart for a normal electron microscope, in order to perform the proposed interferometry experiment^44^.

In summary, the demonstrated spectral interferometry technique uses electron excitation and CL detection to acquire an energy–distance CL map, which can be directly used to extract the time–energy correlation function. The time–energy correlation function is then Fourier–transformed to obtain the two-energy correlation and retrieve the spectral phase and intensity for direct electric field reconstruction. This is achieved by a precise design of a photon source, which uses interaction with the electron beam to generate focused transition radiation that is mutually coherent with the evanescent field of the electron itself. Due to the nature of the designed metamaterial-based photon source, the emitted photon beam is ultrabroadband, and interestingly demonstrates optical excitations which mimic Laguerre Gaussian transverse magnetic optical beams. The presented methodology renders itself useful for a vast range of applications in the fields of ultrafast science, and paves the way towards attosecond electron-based spectroscopy techniques. The presented EDPHS design by itself has applications in the full coherent control of electron induced emissions. In such a way, even few-photon sources would be enough to perform spectral interferometry. There is no apparent need for ultrahigh intensive laser radiation, as far as applications are restricted to linear manipulations of the optical density of states.

## Method

To calculate the electromagnetic response of the sample to the relativistic electron excitation and also the reaction of the scattered field on electron, a numerical self-consistent method has been introduced. The calculations are within the framework of the coupled Maxwell-Lorentz system of equations, and a time-domain finite-differentiation method is used. Within this approach, the electromagnetic fields are defined at discrete spatial points. To simulate the behavior of the fields, Yee-based gridding is exploited^45^. The motion and velocity of the particle is described by the Newton-Lorentz forces as


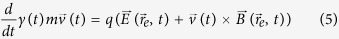



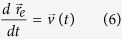


where 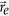 is the electron trajectory, 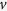 is the electron velocity, *m* electron mass, 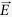 and 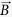 are the scattered electric and magnetic field components. Both fields and particles are defined at discrete time. However, in contrast to the field components, the motion and trajectory of the particle are considered in continuum space^46^.

Classically, electrons are introduced as temporally and spatially localized charge density distributions defined as 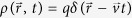, where 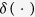 is the Dirac-delta function. However, such a mathematically singular point source leads to an infinite self-energy. In principle, the mass renormalization principle in classical electrodynamics of the Maxwell equations and in quantum electrodynamics has suggested to discard this term, and to attribute the total mass of the electron to what is observed in practice.

The usual approach in particle-in-cell simulations is to map the charges to the grids of the simulation domain. In fact, the interaction of continuum particles with the discrete fields is provided by using interpolation schemes. These interpolation methods include nearest grid points, linear interpolation, quadratic interpolations and so on. Despite their success in plasma physics, simulation of a single relativistic electron and the self-field that it produces, deviates quite heavily from the singular electron self-field, and in some cases, may also lead to spurious forces exerted by the scattered fields on the electron.

An easy approach is to introduce distributions which at the limit lead to a Dirac delta function, such as a Gaussian distribution. I consider a 3-dimensional Gaussian charge distribution at the rest frame, given by


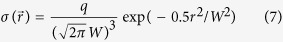


where *q* is the electron charge and *W* is a parameter governing the extension of the charge distribution. It is evident that the total charge is given by 
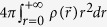
, which is equal to *q*. Moreover, 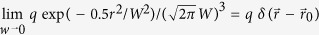. The self-energy at the static field of such an electron source can be obtained as 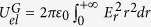 (*E*_*r*_ is the radial component of the electric field and 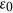 is the vacuum permittivity), which is not infinite in contrast to the case of a singular charge distribution. Computation of the self-energy requires numerical integration. In fact, the self-energy integral can be simplified to:


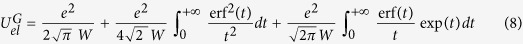


where 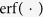 is the error function. Using trapezoidal numerical integration, this value is computed as 
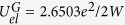
. It can be shown that the electromagnetic mass attributed to such a charge-density distribution is 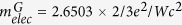. In the laboratory frame, defined by (*ct*′, *x*′, *y*′, *z*′), the current distribution associated with a Gaussian electron distribution moving along the z-axis is given by


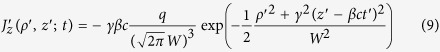


which is introduced into the simulation domain to obtain the scattered electromagnetic field. Here, 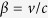 and *c* is the velocity of light. Considering this approach, the relativistic equation of motion for a single electron is written as


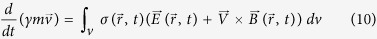


For discretizing eq. (6) in the time domain, the Vay scheme^47^ is used, which leads to more accurate results in comparison with the Boris scheme, and the spurious forces on the electron are minimized.

## Additional Information

**How to cite this article**: Talebi, N. Spectral Interferometry with Electron Microscopes. *Sci. Rep.* **6**, 33874; doi: 10.1038/srep33874 (2016).

## Supplementary Material

Supplementary Information
