# Supplementary material for: Spectral Interferometry with Electron Microscopes
Source: Sci Rep. 2016 Sep 21;6:33874. doi: 10.1038/srep33874 (PMC5030644; doi:10.1038/srep33874)
Supplement: Supplementary Information [file srep33874-s1.pdf]

Supplementing Information for

Spectral Interferometry with Electron Microscopes

Nahid Talebi

Stuttgart Center for Electron Microscopy, Max Planck Institute for Solid State Research,

Heisenbergstr. 1, 70569 Stuttgart, Germany

E-mail:n.talebi@fkf.mpg.de

This PDF file includes:

Supplementary Text
Figs. S1 to S2

Supplementary Text

Using the developed numerical toolbox, the interaction of a relativistic electron with the EDPHS and the sample is simulated. The electron has a Gaussian charge distribution with $W = 5\,\text{nm}$ at an initial velocity of $v_0 = \text{-}0.6953\,c$ along the z-trajectory. Fig. S1 shows the modulation in electron velocity during the interaction, for several distances between the EDPHS and the sample.

The electron-induced polarization in EDPHS (Fig. S2a) causes a focused transition radiation, as described in the main text. Here, the behavior of the radiation and its polarization state is demonstrated more precisely. Fig. S2b shows the temporal behavior of the radiated beam at the focal point. It is apparent that the radiation is in the form of a few-cycle, positively chirped pulse with a broad spectrum.

The transition radiation of the EDPHS is in the form of transverse magnetic (TM_z) mode, with the only nonzero field components given by $E_r, H_\varphi,$ and E_z. The Fourier-transformed field profile of the EDPHS is shown in Fig. S2c, for the E_z and also for the transverse $E_t = \sqrt{E_x^2 + E_y^2}$ components, at the energies 1.5 eV, 2.5 eV, 3.5 eV, and 4.5 eV. Interestingly, the field distribution is very similar to the Laguerre-Gaussian optical beams, with the mode orders increasing at higher photon energies.

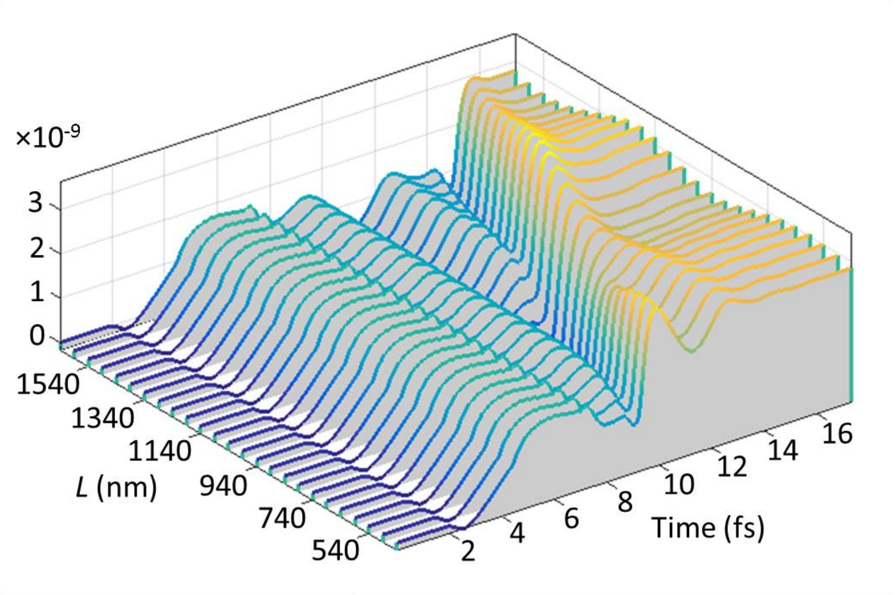

Figure S1. Modulations of the electron velocity versus time and distance between the EDPHS and the sample.

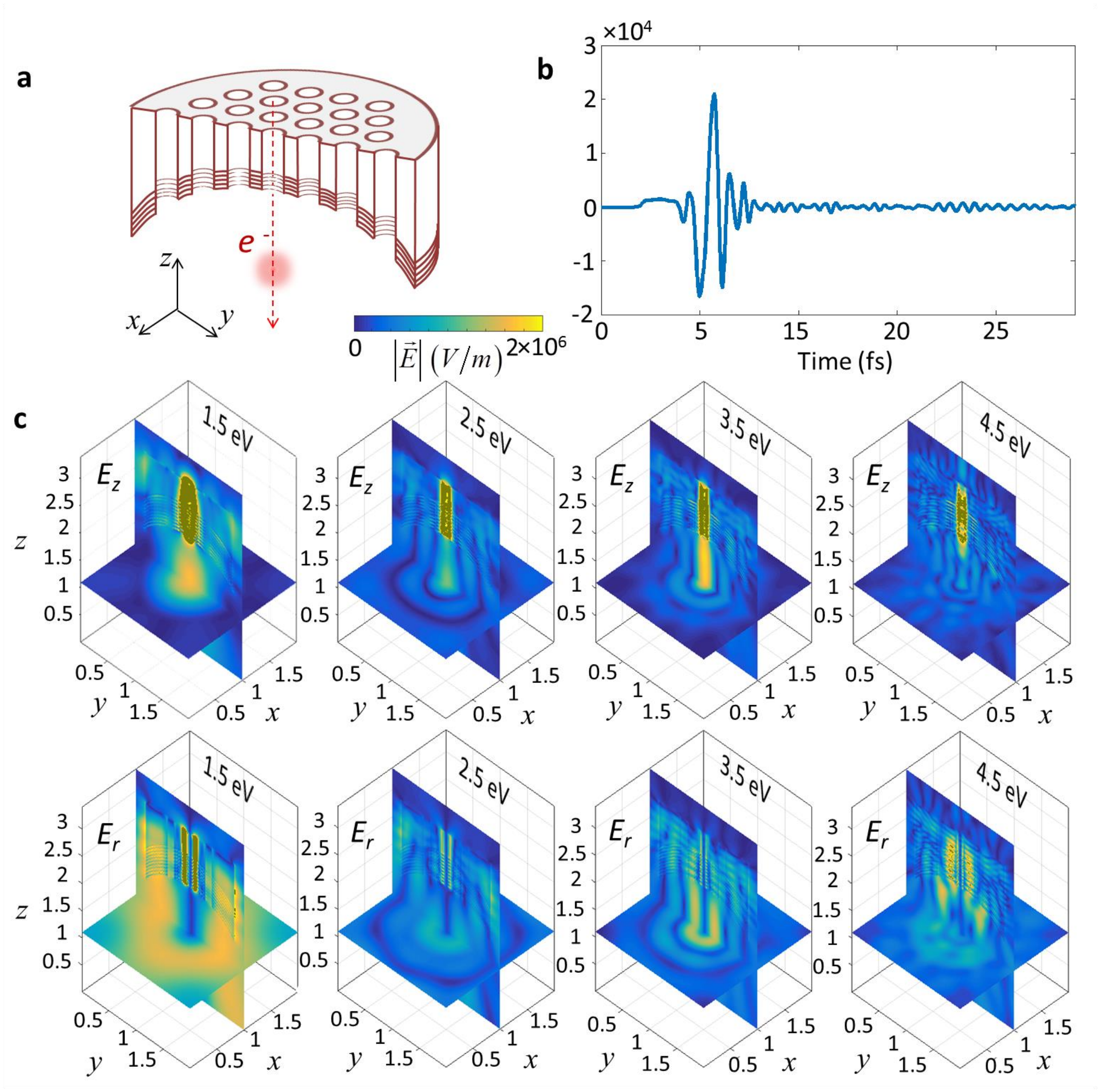

Figure S2. Characterization of the radiation from EDPHS. (a) the EDPHS structure (for more details see the main text), (b) z-component of the electric field at the focal point of EDPHS versus time, (c) spatial distribution of the electric field components E_z and $E_t = \sqrt{E_x^2 + E_y^2}$, at the photon energies depicted at each frame.
